# Microstructure Evolution and Deformation Mechanism of Tantalum–Tungsten Alloy Liner under Ultra-High Strain Rate by Explosive Detonation

**DOI:** 10.3390/ma15155252

**Published:** 2022-07-29

**Authors:** Heng Fu, Jianwei Jiang, Jianbing Men, Xinfu Gu

**Affiliations:** 1 State Key Laboratory of Explosion Science and Technology, Beijing Institute of Technology, Beijing 100081, China; fuheng9999@163.com (H.F.); bitjjw@bit.edu.cn (J.J.); 2 School of Materials Science and Engineering, University of Science and Technology Beijing, Beijing 100083, China

**Keywords:** tantalum–tungsten alloy, explosively formed projectile, ultra-high strain rate, deformation mechanism, dynamic recrystallization

## Abstract

The microstructure evolution and plastic deformation mechanism of a Ta-2.5W liner under the ultra-high-strain-rate conditions generated by the explosive detonation were investigated in this study. For this purpose, a modular soft-recovery apparatus was designed to non-destructively recover the Ta-2.5W explosively formed projectile (EFP) in the ballistic endpoint. The electron backscattered diffraction (EBSD) method was employed to examine the microstructure of the Ta-2.5W liner before and after deformation. The microstructure of the recovered EFP exhibited significant grain refinement with preferred fiber texture. The theoretical computation results showed that the temperature of the EFP was in the range of 0.27–0.65 T_m_. The deformation mechanism of the Ta-2.5W liner forming EFP driven by the detonation is the continuous dynamic recrystallization (CDRX) induced by high strain deformation, rather than the conventional dynamic recrystallization of nucleation and growth. The new grain structures evolve when the low-angle grain boundaries are transformed into the high-angle grain boundaries, and the specific grain refinement mechanism is the progressive rotation of subgrains near pre-existing grain boundaries.

## 1. Introduction

The shaped charge device is a basic explosive system for driving metallic liners to high supersonic velocities using detonation phenomena of a hollow charge which is widely used in defense, military and commercial industries [1]. As one of the key components of the shaped charge device, the material properties and structural parameters of the liner will directly affect the forming state and terminal effect of the projectile [2,3]. The liner exhibits a completely different mechanical behavior and deformation mechanism from the conventional process, including shaped charge jet (SCJ) and explosively formed projectile (EFP).

SCJ means that the classical conical liner with an acute apex angle (<90°) is collapsed symmetrically to form a cumulative jet with a tip velocity of 8–12 km/s, while the velocity of the slug flows is less than 1 km/s [4]. The standoff distance of SCJ generally does not exceed 8 times the charge diameter. The open-angle (120°~180°) conical liner or a spherical one is driven to form an EFP, also known as self-forging fragments (SFF), which is used to attack long-range targets [5,6]. Although the forming process of an EFP is very different from that of SCJ [7], both are typical examples of extreme plastic deformation of metal liners driven by explosive detonation. Under the high shock pressure (>20 GPa) and ultra-high strain rate (>10^5^ s^−1^) generated by detonation, the strain in the liner reaches between 300% and 1000%, while accompanied by a sharp increase in temperature [8].

It has been nearly 130 years since the discovery of the shaped charge device, and its high-speed development began with the successful application in the Second World War. Many researchers from all over the world have made important contributions to the field of shaped charge, but the great majority of the research is based on the experimental phenomena with empirical or semi-empirical results [2,3,9,10]. Due to the extreme loading and deformation conditions, the actual evolution of the liner material is directly treated as fluid [1,2,3]. At present, the power performance of the shaped charge projectile is already reaching the bottleneck, and it is difficult to achieve further improvement by purely macro-structural-parameter modulation. Despite a small number of exploratory studies on microscopic aspects since the 1990s, the understanding of the specific mechanical behavior and control mechanisms of liner materials is still unclear and incomplete [11].

To explore the mechanical behavior and deformation mechanism of SCJ, many researchers have systematically studied the microstructure of SCJ liner materials before and after deformation. Murr and co-workers examined the microstructure in jet fragments of Cu, Mo and Ta, and the results indicated that dynamic recrystallization (DRX) is the main deformation mechanism in SCJ liners under extreme deformation conditions [12,13,14,15]. The dislocation-free cells commonly present in the recovered slug of electroformed Cu liners have further confirmed that dynamic recrystallization occurs during the deformation process [16]. Yang et al. observed the melting of electroformed Ni liner and believed that the increase in temperature led to the production of DRX [17]. Wang et al. suggested that the high density of dislocation walls consisting of parallel dislocation lines inside the severely elongated and refined grains in pure-tungsten slug and mortars is a consequence of the concentration of dislocations through a single slip system due to the ultra-high strain rate [18,19]. Jiang et al. had observed significant dynamic recrystallization in the recovered slug of a CP-Ti liner, while the α→β phase transformation occurred via a martensitic mechanism [20]. In general, it demonstrates that the liner materials of SCJ exhibit a wide range of DRX phenomena under the explosive detonation.

With the development of precision guidance and weapon equipment technology, EFP shows great potential in new light systems such as intelligent ammunition and small unmanned air vehicles (UAV), which can effectively achieve miniaturization, long-range attack and efficient damage. In recent years, some research has also been performed on the liner material for EFP. Murr observed the existence of refined grains in a pure-Ta EFP, but the grain structure was not identical to that formed by DRX in SCJ [21]. Dense Neumann bands consisting of deformation twins have been observed in an Fe EFP by Pappu et al. [22]. The dynamic response and microstructural changes in a Cu EFP were investigated by Liu et al. and the results showed that complete DRX occurred in the recovered projectile [23]. It shows that different microstructures will be generated during the EFP-forming process.

Tantalum and tantalum–tungsten alloys are highly anticipated as the main candidates for EFP liners at the current stage due to their high density, high melting point, excellent ductility and formability. The basis for effective regulation of the macro properties of EFP lies in the accurate understanding of the deformation mechanism in the liner material. However, the specific deformation mechanism of the tantalum–tungsten alloy liner in the formation of EFP driven by explosive detonation is still unclear. Therefore, the investigation of the microstructure evolution of the tantalum–tungsten alloy liner will contribute to the understanding of the mechanical behavior and deformation mechanism of the body-centered cubic materials involved in the formation process of EFP. Furthermore, it will be beneficial for guiding the structural design of the liner and improving the penetration performance of EFP.

In the present study, a modular soft-recovery apparatus for hypervelocity kinetic penetrators is designed and applied to recover a Ta-2.5W EFP at the ballistic end point safely and non-destructively. A comparison of the microstructure of a tantalum–tungsten alloy liner before and after deformation driven by the explosive detonation was performed using electron backscattered diffraction (EBSD). Quantitative analysis of the temperature rise of the recovered projectile was performed by using theoretical calculations. Furthermore, the deformation mechanism of tantalum–tungsten alloy liner in the EFP-forming process is discussed.

## 2. Experimental Section

**Figure 1:**
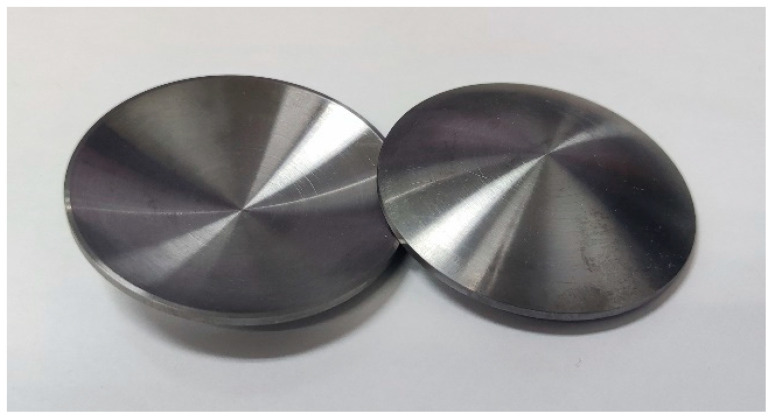
Photograph of the Ta-2.5W liner.

The tantalum–tungsten alloy used in this study is Ta-2.5W which was prepared by electron beam melting. Chemical analysis results revealed that the impurity content was 0.0066% Nb, 0.001% Mo, 0.0015% N, 0.007% O and 0.001% Si by weight percent. The original EFP liner, as shown in Figure 1, is a spherical structure with equal thickness; both the inner and outer curvature radius are 58.55 mm, and the thickness is 1.5 mm.

**Figure 2:**
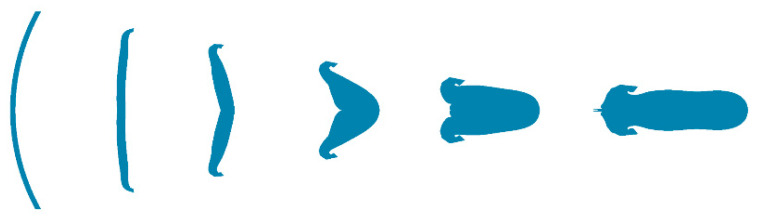
The collapse of a Spherical liner and the formation process of a rod-shaped EFP.

Figure 2 schematically illustrates the collapse of a spherical liner and the formation process of a rod-shaped EFP; the liner is collapsed and turned outward along the axis by the high shock pressure generated by detonation. The central part of the liner moves at a relatively high speed and gradually forms the “head” section, while the other parts follow behind and form the “tail” section of the projectile. As the edge of the liner gradually accumulates inward, the projectile is stretched and deformed under the action of velocity gradient until a stable rod-shaped EFP is formed. This transformation of the liner is completed in less than 200 μs at ultra-high-strain-rate conditions.

The key for microstructure characterization of the deformed liner material rests on the reliable recovery of the projectile in its ballistic ending point. The EFP should be quenched in a sufficiently short time to avoid microstructure changes caused by delayed quenching, in consideration of the high temperature in the projectile. Due to the extremely high flight speed, conventional recovery methods make it difficult to safely decelerate and non-destructively recover the EFP [24]. Such a method as water recovery may easily lead to further plastic deformation or even fracture and fragmentation of the projectile during the penetration process.

To recover the hypervelocity Ta-2.5W EFP non-destructively in a limited space, a modular soft-recovery device was designed. The design principle of the soft-recovery apparatus is on the basis of the kinetic model proposed by Allen et al. [25,26] for the penetration of a projectile into low-density media. The length of each soft medium can be considered according to the density of the soft media and the space of the experimental field. However, it must be ensured that the resistance of the projectile at the moment of incidence is less than the yield strength of the liner material. The final medium used may be water or another suitable liquid for cooling, thus enabling the quenching of the projectile.

**Figure 3:**
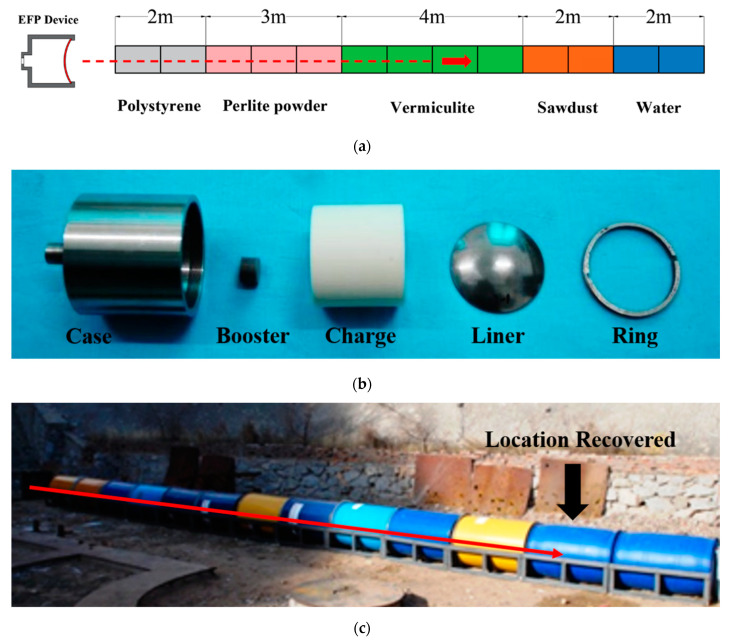
(**a**) Schematic diagrams of the soft-recovery apparatus with an arrow depicting the projectile’s path; (**b**) components of the EFP device and (**c**) photograph of the setup of the recovery experiment.

Figure 3a shows the schematic diagram of the soft-recovery apparatus. The medium was laid with 2 m of polystyrene foam (30 kg/m^3^), 3 m of perlite powder (80 kg/m^3^), 4 m of expanded vermiculite (160 kg/m^3^), 2 m of sawdust (300~350 kg/m^3^) and 2 m of water (1000 kg/m^3^) each, in sequence from the entrance. Figure 3b shows a photograph of the components of the EFP device. The charge is a JH-2 high explosive with a density of 1.71 g/cm^3^. The charge diameter is the same as the liner diameter, and the length-to-diameter ratio of the charge is 0.866. Detonation is performed at the central point in the end of the charge. Figure 3c shows the setup of the recovery experiment. The Ta-2.5W EFP was successfully recovered in the water after the experiment finished. The position of the recovered projectile is shown by the arrow in Figure 3c.

**Figure 4:**
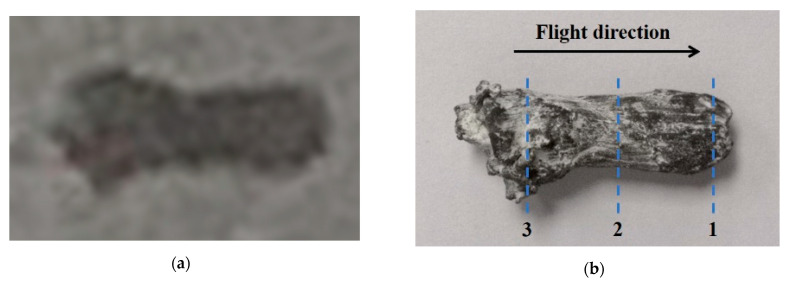
(**a**) X-ray photograph of EFP forming state at 300 μs and (**b**) the macro photograph of the recovered projectile. The flight direction indicates the head and tail of the recovered projectile. The observation direction is along the axial direction of the projectile, which is parallel to the flight direction as shown in (**b**).

Figure 4 shows the shape comparison of Ta-2.5W EFP before and after soft recovery. The forming state of Ta-2.5W EFP at the moment of 300 μs was photographed using pulsed X-ray as shown in Figure 4a. The flight velocity of the projectile is about 2100 m/s. The photograph of the recovered T-2.5W EFP is shown in Figure 4b. The recovered projectile is approximately rod-shaped, with no collision marks on the surface and necking to some extent in the middle part of the projectile. The overall shape of the recovered projectile is consistent with that in Figure 4a, indicating that the EFP did not undergo plastic deformation during the deceleration of the soft media.

The soft medium attached to the surface of the recovered EFP was removed by ultrasonic cleaning. Then, the projectile was cut using electrical discharge machining (EDM) in the perpendicular longitudinal axis, and the position of the cut sample is shown in Figure 4b. The areas 1, 2 and 3 represent the head, middle and tail sections of the EFP, respectively. The observation direction is the axial direction of the EFP, which is consistent with the flight direction in Figure 4b. The samples were ground using the conventional mechanical method and electrolytically polished in 5% perchloric acid solution for 50 s at 27.8 V. The samples were analyzed by electron backscattered diffraction (EBSD) using a TESCAN electron microscope. The scanning step size was 0.5 μm and the phase was selected as tantalum. Data acquisition and processing were performed with the OIM Analysis software.

## 3. Results

**Figure 5:**
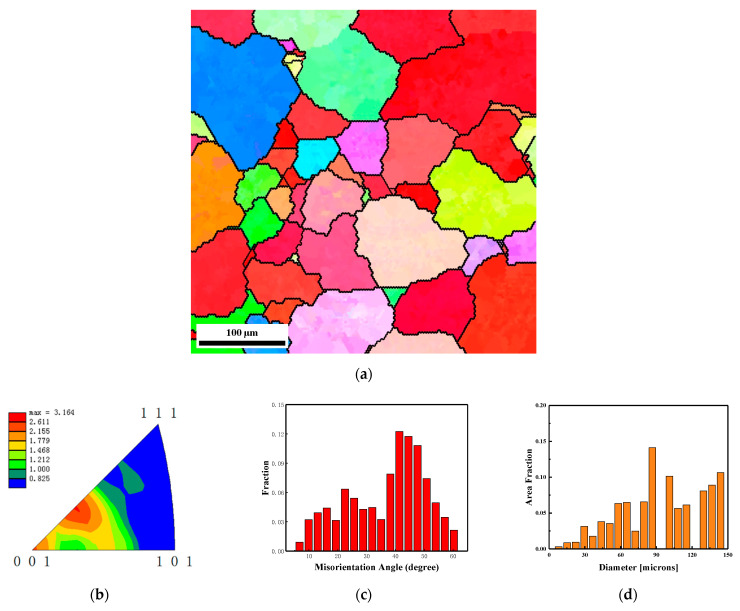
EBSD results of Ta-2.5W liner before the explosive detonation. (**a**) microstructure colored by inverse pole figure and the black lines indicate high grain boundary with misorientation angle larger than 15°, (**b**) texture shown by inverse pole figure of normal direction, (**c**) boundary misorientation distribution and (**d**) grain size distribution.

Figure 5 shows the EBSD results of Ta-2.5W liner before the explosive detonation. As Figure 5a shows, the microstructure of the liner in the initial state is relatively homogeneous which composed of equiaxed crystals. The inverse pole figure of this area in Figure 5b shows there is an obvious texture in the original microstructure of the liner, and the preferred grain orientation of the normal direction is close to parallel to the <114> direction. Figure 5c shows the statistic of the misorientation angle, and the result shows that most grain boundaries are grain boundaries (HAGBs), and the percentage is more than 90%. The grain size distribution is shown in Figure 5d and the average grain size is about 45 μm. It can be seen from Figure 5c that the percentage of grains with a diameter larger than 30 microns in the liner is about 95%.

**Figure 6:**
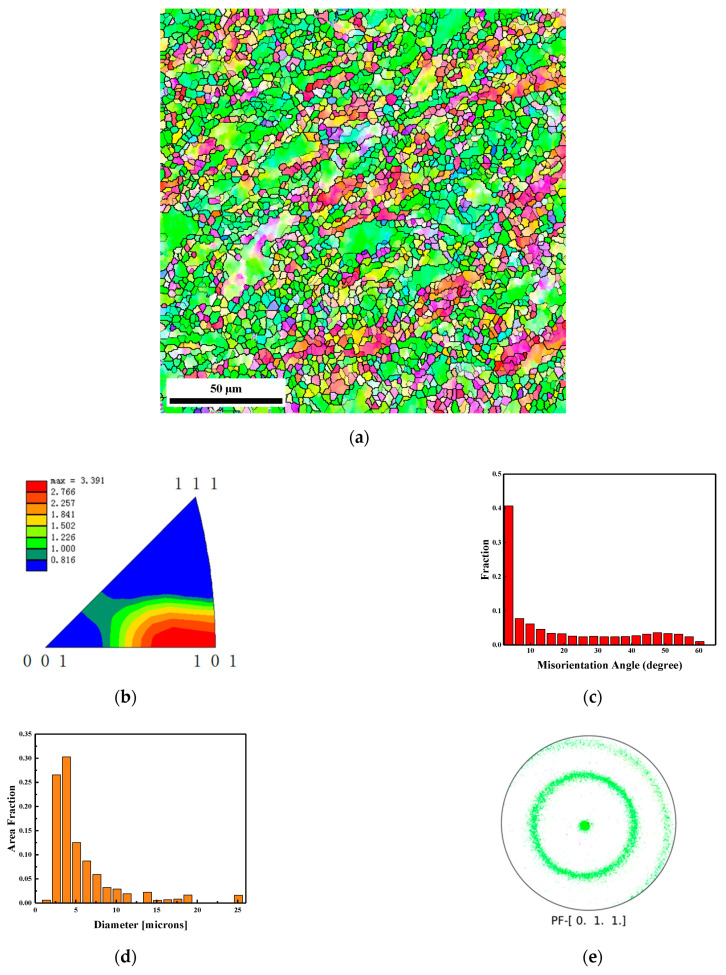
EBSD results of the head section marked by area 1 in the recovered EFP. (**a**) microstructure colored by inverse pole figure and the black lines indicate high grain boundary with misorientation angle larger than 15°, (**b**) texture shown by inverse pole figure of normal direction, (**c**) boundary misorientation distribution, (**d**) grain size distribution and (**e**) {011} pole figure.

Figure 6 shows the EBSD results of the head section marked by area 1 in the recovered EFP. The microstructure of the head section in the recovered projectile is shown in Figure 6a, where the grains are obviously refined after extreme plastic deformation, while a few deformed large grains are still present. Figure 6b shows the inverse pole figure of the same area in Figure 6a. The result shows that the preferred grain orientation in the head section is mainly in the <101> direction, which is greatly different from the original microstructure in the liner. The statistic of misorientation angle in Figure 6c shows that a great amount of misorientation accumulated in the microstructure of the head section. The proportion of low-angle grain boundaries (LAGBs) is approximately equal to 65%. Figure 6d shows the grain size distribution; the result shows that the maximum and average grain size is about 25.1 μm and 3.4 μm, respectively. The fraction of the refined grains whose diameter is less than 5 μm in the head section of the EFP is about 57%. The <101> fiber texture, as confirmed by the {011} pole figure, is generated from the uniaxial elongation of the projectile during the denotation, and the grain is refined during the deformation.

**Figure 7:**
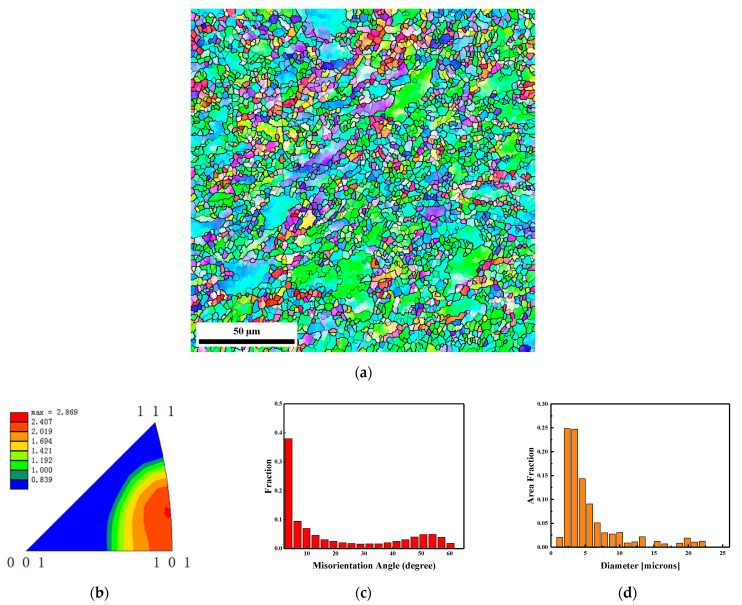
EBSD results of the middle section marked by area 2 in the recovered EFP. (**a**) microstructure colored by inverse pole figure and the black lines indicate high grain boundary with misorientation angle larger than 15°, (**b**) texture shown by inverse pole figure of normal direction, (**c**) boundary misorientation distribution and (**d**) grain size distribution.

Figure 7 shows the EBSD results of the middle section marked by area 2 in the recovered EFP. As is shown in Figure 7a, the microstructure of the middle section of the projectile is similar to that of the head, which is also a mixture of refined grains and deformed large grains. The corresponding inverse pole figure in Figure 7b indicates that the middle section of the projectile also has a texture in the <101> direction, with a gradual deflection to <111>. According to the statistics of misorientation angle in Figure 7c, it can be seen that the percentage of HAGBs in the middle section is slightly increased than the one in the head section of the recovered EFP. The grain size distribution in Figure 7c shows that the maximum and average grain size of the middle section in the EFP are about 22.0 μm and 3.4 μm. Both of them are slightly reduced compared to the projectile head region shown in Figure 6, while the percentage of grains of less than 5 µm in diameter increases to 66%.

**Figure 8:**
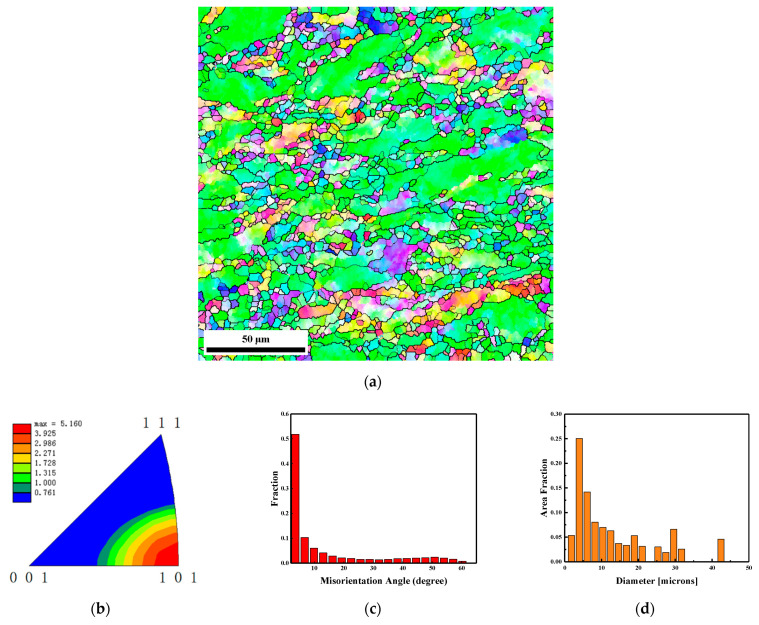
EBSD results of the tail section marked by area 3 in the recovered EFP. (**a**) microstructure colored by inverse pole figure and the black lines indicate high grain boundary with misorientation angle larger than 15°, (**b**) texture shown by inverse pole figure of normal direction, (**c**) boundary misorientation distribution and (**d**) grain size distribution.

Figure 8 shows the EBSD results of the tail section marked by area 3 in the recovered EFP. As Figure 8a shows, more large grains remain in the microstructure of the tail section of the EFP compared with Figure 6 and Figure 7. It may be attributed to the different strains at different locations of the liner during the EFP formation process. The inverse pole figure shown in Figure 8b indicates that the preferred grain orientation of this region is also in the <101> direction, while having the maximum texture strength. As shown in Figure 8c, the tail section of the projectile features the highest percentage of LAGBs. As is shown in Figure 8d, the maximum and average grain size of the middle section in the EFP is about 42.4 μm and 4.4 μm. Additionally, the percentage of refined grains less than 5 μm reached only 33%.

Overall, the EBSD results show that significant grain refinement took place in the Ta-2.5W liner during the EFP-forming process. It was transformed from a uniform original microstructure to a mixture of fine equiaxed grains and residual deformed large grains. The average grain size was refined to about 1/10 to 1/15 of that before the explosive loading deformation. In contrast to the liner before detonation, a new fiber texture developed in the recovered EFP, which transformed the preferred grains’ orientation from parallel close to the <114> direction to the <101> direction. At the same time, the percentage of LAGBs increased sharply. The microstructure evolution of different areas in the EFP were not identical. The difference may be due to the gradient strain, which caused various deformation levels in different areas. In addition, no twins were observed in the recovered EFP. Probably, the twins were hindered or annihilated by the rapid heating effect owing to the ultra-high-strain-rate deformation.

The deformation mechanism of the Ta-2.5W liner driven by explosive detonation is initially considered to be DRX according to the refined grains observed in the recovered EFP. It is noteworthy that the grain sizes of the fine equiaxed crystals in different areas of the recovered projectile are basically the same, and there seems to be no difference in the nucleation and growth stages. Anyway, the evolution of the microstructure in the liner material is closely related to the intense heat that is generated by the ultra-high-strain-rate plastic deformation. In the following section, the temperature increment of the Ta-2.5W liner during the formation of the EFP will be quantitatively analyzed combined with theoretical calculations, so that the deformation mechanism can be further discussed.

## 4. Discussion

### 4.1. Temperature Rise in the Formation Process of EFP

The velocity of the shock wave generated by the explosive detonation was extremely high, and the absolute time of interaction with the liner micro-element was less than 1 μs. The temperature rise of the metal liner under the action of a shock wave can be regarded as a transient process. The shock temperature $T_{s}$ can be calculated by the following formula [27]

(1) $$T_{s}=T_{0}\exp\left[\frac{\gamma_{0}}{V_{0}}\left(V_{0}-V_{1}\right)\right]+P\frac{\left(V_{0}-V_{1}\right)}{2C_{V}}+\frac{\exp\left(-\frac{\gamma_{0}}{V_{0}}V_{1}\right)}{2C_{V}}\int_{V_{0}}^{V_{1}}P\cdot \exp\left(\frac{\gamma_{0}}{V_{0}}V\right)\left[2-\frac{\gamma_{0}}{V_{0}}\left(V_{0}-V\right)\right]dV$$

where $T_{0}$ is the initial temperature of the liner; $\gamma_{0}$ is the Mie–Gruneisen parameter of the liner material; *P* is the pressure of the shock wave; $C_{V}$ is the heat capacity of the liner material; $V_{0}$ is the initial specific volume of the liner material; and $V_{1}$ is the specific volume behind the shock wave. $V_{1}$ can be deduced from the relationship between the parameters of the shock wave.

(2) $$P=\frac{c_{0}{}^{2}\left(V_{0}-V_{1}\right)}{{\left[V_{0}-\lambda \left(V_{0}-V_{1}\right)\right]}^{2}}$$

(3) $$V_{1}=\frac{c_{0}{}^{2}}{2\lambda^{2}P}\left[\sqrt{1+\frac{4\lambda PV_{0}}{c_{0}{}^{2}}}+\frac{2\lambda \left(\lambda -1\right)PV_{0}}{c_{0}{}^{2}}-1\right]$$

where $c_{0}$ and λ are Hugoniot constants for the liner material.

After the shock wave loading, the unloading of the rarefaction wave is considered as an isentropic process. The residual shock temperature $T_{r}$ of the liner can be expressed by

(4) $$T_{r}=T_{s}\exp\left[-\frac{\gamma_{0}}{V_{0}}\left(V_{0}-V_{1}\right)\right]$$

**Figure 9:**
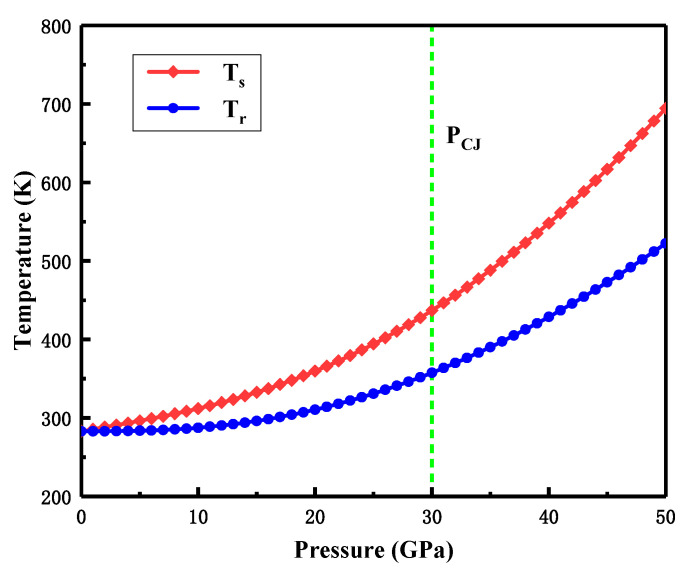
Temperature rise for the Ta-2.5W liner caused by shock wave. P_CJ_ indicates the shock pressure generated by the detonation of JH-2 explosives.

For the Ta-2.5W alloy used in the present study, $T_{0}=283\;K$, $\gamma_{0}=1.7$, $C_{V}=130\;J/\left(\mathrm{kgK}\right)$, $c_{0}=3.31\;\mathrm{km}/s$ and λ=1.3. The results of the temperature rise of the T-2.5W liner caused by the shock wave are shown in Figure 9. Both $T_{s}$ and $T_{r}$ rose with the increase in shock pressure. The result shows that the residual shock temperature of Ta-2.5W liner was 358 K, under the shock pressure generated by the detonation of JH-2 explosive which was about 30 GPa.

Subsequently, the temperature rise associated with plastic deformation can be calculated by the following equation

(5) $$\Delta T=\frac{\beta }{\rho C_{P}}\int_{0}^{\varepsilon }\sigma d\varepsilon$$

where *σ* is the stress; ε is the strain; ρ is the density of the liner material; and β is the conversion factor, which is generally taken as 0.9 for metallic materials. For the stress *σ*, the Johnson–Cook model [28], which is suitable to characterize the strength behavior of the material at large strains, high temperatures and high strain rates, is chosen to describe

(6) $$\sigma =\left(A+B\varepsilon_{p}{}^{n}\right)\left(1+C\ln\frac{\dot{\varepsilon }}{{\dot{\varepsilon }}_{0}}\right)\left(1-T^{*}{}^{m}\right)$$

where A, B, n, C and m are the material’s parameters measured by the experiments; $\dot{\varepsilon }$ is the strain rate; ${\dot{\varepsilon }}_{0}$ is the reference strain rate; and $T^{*}$ is the homologous temperature which can be expressed by

(7) $$T^{*}=\frac{T-T_{r}}{T_{m}-T_{r}}$$

where T is the current temperature; $T_{m}$ is the melting temperature; and $T_{r}$ is the residual shock temperature, that is, the onset of plastic deformation.

**Figure 10:**
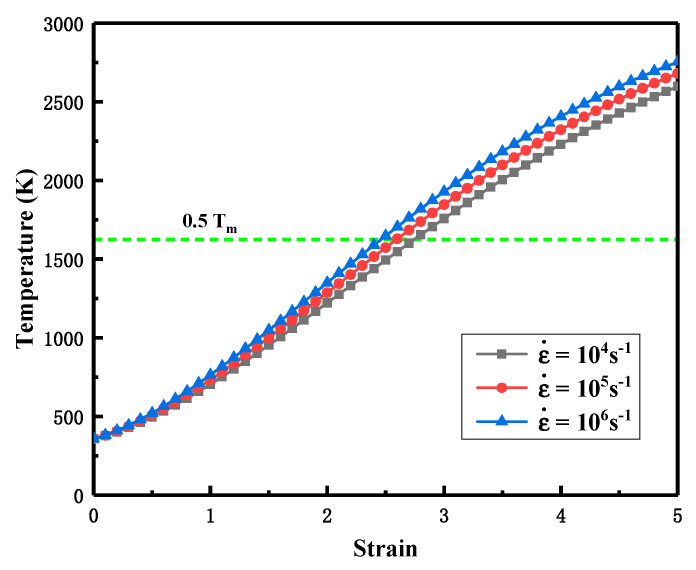
The temperature versus strain of Ta-2.5W at the strain rates of 10^4^, 10^5^, and 10^6^ s^−1^. T_m_ indicates the melting point of Ta-2.5W.

Based on the literature [29], the material parameters in the Ta-2.5W for the Johnson–Cook model were obtained and are listed in Table 1. Figure 10 shows the calculated temperature versus the strain of Ta-2.5W at different strain rates. Three strain rates, respectively, 10^4^ s^−1^, 10^5^ s^−1^ and 10^6^ s^−1^, were selected. The notation T_m_ in the figure indicates the melting point of Ta-2.5W. Detailed strain data of the liner during the EFP forming process were acquired from numerical simulations with the finite element software Ansys Autodyn. Because of the symmetry of the EFP device, a quarter geometry model was used for the numerical simulations. The Lagrange algorithm was employed to construct the finite element models with hourglass control. The model was discretized with 8-node hexahedral solid elements. The explosion expansion process of high-energy explosive was described by the Jones–Wilkins–Lee (JWL) equation of state. The Johnson–Cook model and the SHOCK equation of state were used to describe the strength behavior of metallic components. The parameters for the Ta-2.5W liner are listed in Table 1. The detailed parameters of the JH-2 explosive and 45# steel case are from the literature [30]. Gaussian points were used to track the Lagrange elements at the center and edge of different areas of the EFP in Figure 4b.

**Figure 11:**
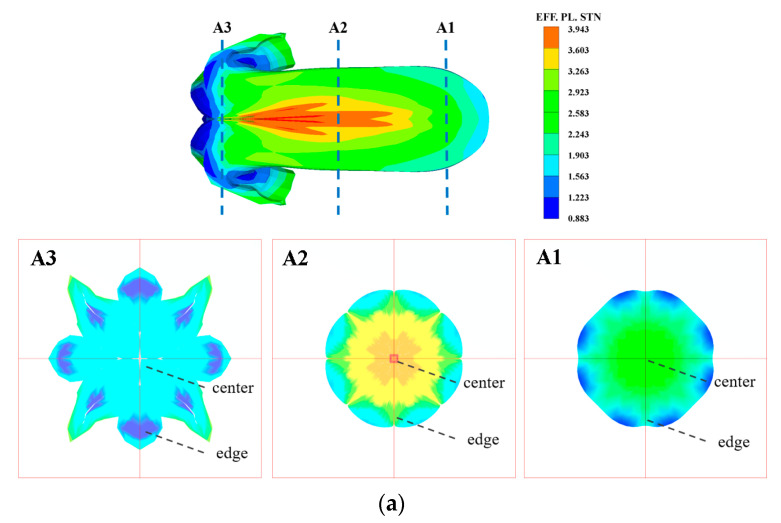

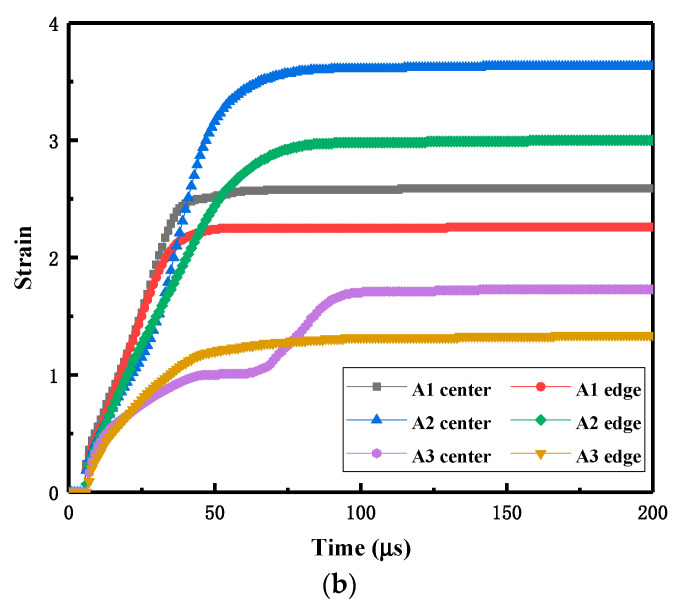
Numerical simulation results of Ta-2.5W EFP by Ansys Autodyn. (**a**) Effective plastic strain distribution of EFP. (**b**) Strain versus time plots at centers and edges of different areas marked in (**a**). The slice planes A1, A2 and A3 are corresponding to the areas 1, 2 and 3 in the recovered projectile shown in Figure 4b, respectively.

Figure 11 shows the numerical simulation results of Ta-2.5W EFP. As can be seen from Figure 11a, the strain in the central area of the EFP is the largest, while the smallest is at the tail area. The statistical results of the strain–time history curves in Figure 11b show that there are temporal differences in the dynamic response of the liner elements to the shock wave. Because of the inward closure of the liner during the deformation process, the strain on the recovered projectile specimen decreases gradually from the center to the edge.

Table 2 shows the calculated results of temperature in different areas of the recovered EFP. Among them, the lower and upper limits of temperature were carried out with strain rate 10^4^ s^−1^ and 10^6^ s^−1^, respectively. According to the calculations’ results in Table 2, the temperature range of the EFP is about 0.27~0.65 T_m_. The highest temperature in area 2 was about 0.54~0.65 T_m_, and the lowest temperature in area 3 was about 0.27~0.36 T_m_. The temperature in area 1 was in between the previous two, reaching 0.42~0.51 T_m_. Generally, only the deformation temperature at the middle section (area 2) of the EFP completely reached the hot working condition (T > 0.5 T_m_), while the head (area 1) and tail sections (area 3) were in the warm/cold working condition (T < 0.5 Tm). Although the deformation temperature of the projectile head (area 1) failed to fully reach more than half of the material-melting temperature, its microstructure demonstrated a complete recrystallization similar to that of the middle section (area 2). It implies that temperature is not the main factor to influence the microstructure evolution of the Ta-2.5W liner during deformation at ultra-high strain rates.

As competing mechanisms during material deformation, twinning and dislocation slip often occur at different strain rates and temperature conditions. The deformation twinning in body-centered cubic metals usually takes place in the loading conditions of low temperatures and high strain rates. Unlike the observation of twins in tantalum under a plane shock wave, reported by Meyers [31,32], twins were not observed in the recovered EFP of this study. Although, the plane shock wave is also generated by the explosive, but the strain in the flying plate can be negligible compared to that in the liner. According to the results of the quantitative analysis of the liner temperature, it is clear that the main factor influencing the material temperature is the extreme plastic deformation. The shock wave alone has little effect on the temperature increase in the material. The presence of twins was likewise not observed in pure Ta SCJ with much higher strain [33]. It is the adiabatic temperature rise caused by the rapid deformation of the liner at ultra-high strain rates that prevents the formation of twins.

For metals, the stacking fault energy directly determines the width of the stacking fault which influences the level of perfect dissociation into partial dislocations. As a stacking fault energy material, the perfect dislocations in Ta-2.5W alloy are much more prone to slip, climb and cross-slip. In contrast, the reduction in stored energy during dynamic recovery is achieved by dislocation slip. The rapid deformation at ultra-high strain rates further increases the rate of dynamic recovery (DRV), resulting in the formation of subgrains in the original grains. This explains the large number of low-angle misorientations accumulated in the recovered EFP. Hence, the rearrangement and annihilation of dislocations in the Ta-2.5W alloy can easily occur through rapid DRV, which prevents the accumulation of sufficiently high dislocations for nucleation.

### 4.2. Deformation Mechanism and Grain-Refinement Process

The conventional DRX is that the dislocation density increases to a high level during the deformation process, and new grains begin to nucleate when the accumulated dislocation difference in density reaches sufficiently high, followed by the migration of HAGBs. It is well known that this process, also known as discontinuous dynamic recrystallization (DDRX), is considered a two-step phenomenon. DDRX is essentially a thermal process controlled by lattice diffusion and occurs during straining at the temperature above about half the melting point [34,35].

The calculated temperature results in Table 2 show that the generation of fine grains in the recovered EFP involves both thermal and non-thermal processes. In fact, the refined grains appear in the whole volume, not only in the middle section where the temperature reaches the thermal-processing conditions. Thus, it can be determined that the deformation of the Ta-2.5W liner may not be controlled by DDRX. Such a phenomenon of new grain structures that evolve during straining at both hot and cold temperatures is much more similar to continuous dynamic recrystallization (CDRX).

CDRX is considered as a one-step phenomenon, which is essentially a continuous reflection induced by strain [36]. In CDRX, the mechanism of new grain formation lies in the continuous evolution of the subgrain structure, which is quite different from the nucleation caused by the dislocation density difference in conventional DDRX. The constant increase in misorientation and the continuous decrease in grain size of the subgrains during plastic deformation are the essential characteristics of CDRX observed from different materials [37,38,39,40,41,42,43].

**Figure 12:**
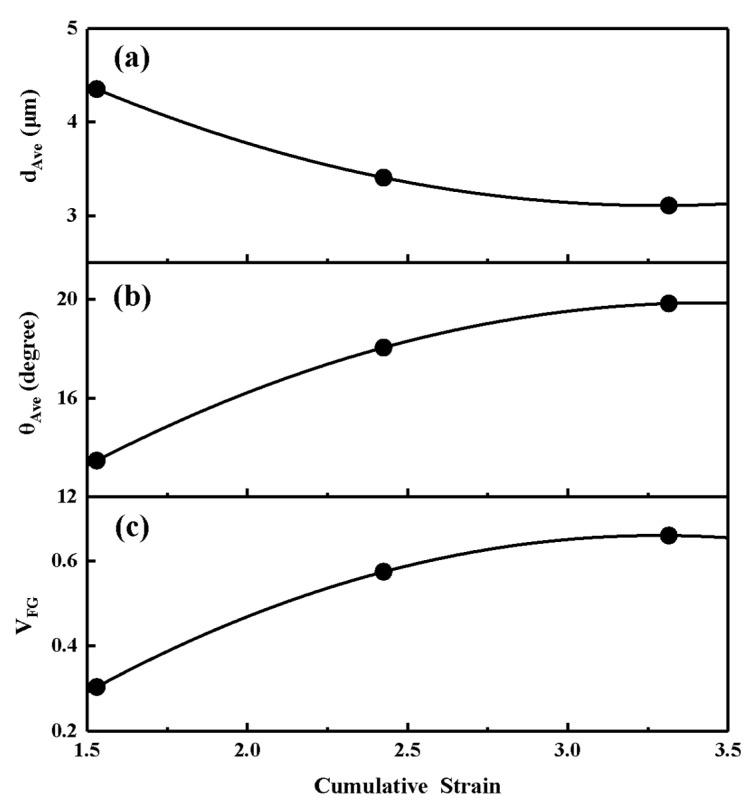
Effect of strain during the formation process of Ta-2.5W EFP on (**a**) average grain size *d*_AVE_, (**b**) average boundary misorientation *θ_AVE_* and (**c**) volume fraction of fine grains *V_FG_*. Cumulative strains are calculated by averaging the strains at the center and edge of the region from the numerical simulation.

Figure 12 illustrates the effect of strain on the average grain size *d*_AVE_, the average boundary misorientation *θ*_Ave_, and volume fraction of fine grains *V*_FG_. Taking into account the approximately linear decreasing strain from the center to the edge of the sample area in EFP, the average value of the strain data was taken as the cumulative strain. As shown in Figure 12a, a decrease in the average grain size takes place with increasing strain. This trend of continuous decrease in the grain size can commonly be found in the CDRX process at large strain states [39,40,41]. The peaks related to the refined grains formed by recrystallization can be observed on the histograms of grain size distribution from all strains (from Figure 8d to Figure 6d and then to Figure 7d). The uniformity of the microstructure continues to develop as the strain increases. At a strain of 1.5, the appearance of refined grains shown in Figure 8d indicates that the deformation of the EFP is already above the critical strain that is necessary to start the DRX.

The data in Figure 12b clearly demonstrate the average misorientation increased with strain. In the present case, the maximum value of the average misorientation is about 20°, which is similar to that reported for CDRX in ferritic stainless steel under severe plastic deformation [42]. The density of HAGBs increases with deformation for all strains in Figure 12b. Inspection of these data shows that further straining results in a gradual decrease in the density of LAGBs. LAGBs continuously transform into HAGBs, providing a continuous increase in the proportion of HAGBs. Therefore, the peak associated with HAGBs can be observed to gradually increase on the histograms of misorientation distribution with strain (from Figure 8c to Figure 6c and then to Figure 7c).

Figure 12c shows the fraction of refined grains increases gradually with deformation. The percentage of refined grains increases with strain, which is the reason for the constant decrease in the average grain size. It is seen that an increase in the fraction of refined grains induced by deformation is the main process of microstructural evolution at strain >1.5. In the strain interval investigated, the fraction of refined grains can be approximated by a linear function of strain, which is consistent with that in the average misorientation. At the early stage of deformation, the high percentage of LGBSs is associated with the formation of a well-developed subgrain structure. The dislocations introduced by the deformation are rapidly rearranged and transformed into dislocation sub-boundaries by the greatly enhanced dynamic recovery due to the ultra-high strain rate and high stacking fault energy of Ta-2.5W. LAGBs continuously transform into HAGBs providing a continuous increase in the proportion of the fraction of new grains in a wide range of temperatures [41,43].

**Figure 13:**
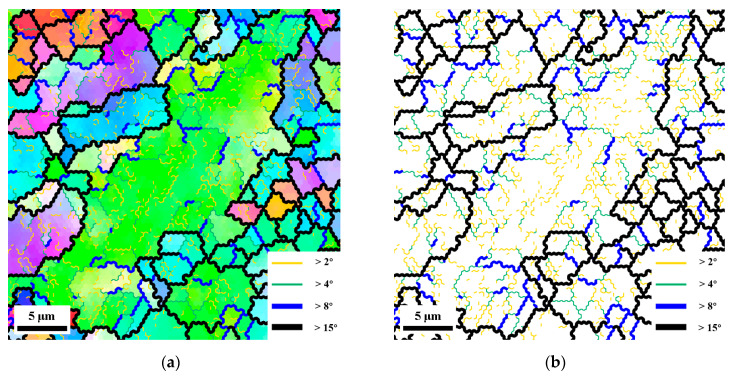
(**a**) Microstructure and (**b**) grain boundary distribution of residual deformed large grains in area 2. Thin yellow lines correspond to boundaries of misorientation > 2°, thin green lines > 4°, thick blue lines > 8°, and thick black lines > 15°.

The above trends in microstructural parameters with strain are similar to those of other materials in which CDRX occurs. The variation in the misorientation will be further discussed below. Figure 13 shows the microstructure and corresponding grain boundary distribution of the residual large grains in area 2. The thin yellow line, thin green line, thick blue line and thick black line represent the grain boundaries of 2~4°, 4~8°, 8°~15° and more than 15°, respectively. From the microstructure in Figure 13a, it can be seen that there are many deformation substructures inside the residual large grains. The LAGBs constitute the necessary geometric boundaries for these substructures which are present in the thermally processed regions. It means that new grains are formed by deformation substructures via LAGBs transforming into HAGBs. It is obvious from Figure 13b that the misorientation of the low-angle grain boundaries within the residual large grains gradually increases from inside to outside. The centrally located substructures are basically formed by the LAGBs of 2~4°. The LAGBs of 4~8° are mainly found at the edge of large grains inside, while the LAGBs of 8~15° are mostly connected with HAGBs, and these features are originated from the strain accommodated between neighboring grains. In addition, the orientation of the recrystallized grains at the margin of the large grain is clearly different from the <101> direction in Figure 13a. It indicates that the LAGBs within the residual large grains at high temperatures are gradually increasing from the outside to the inside, rather than a uniform process of simultaneous increase [38,44]. Thus, it can be determined that the transition from LAGBs to HAGBs during deformation is achieved by the progressive rotation of subgrains within parent grains.

**Figure 14:**
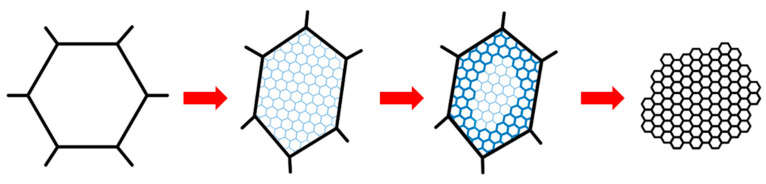
Schematic illustration of the grain-refinement process of Ta-2.5W liner driven by explosive detonation.

In summary, the plastic-deformation mechanism of Ta-2.5W liner under the ultra-high strain rate generated by the explosive detonation is CDRX, and the transformation of LAGBs to HAGBs is performed by the progressive lattice rotation near grain boundaries. Based on the above experimental results and related analysis, a model for the grain refinement of the deformation of Ta-2.5W driven by explosive detonation is developed, as shown in Figure 14. A large number of dislocations introduced by ultra-high strain rate deformation inside the Ta-2.5W grains are rapidly annihilated and rearranged by the enhanced dynamic recovery. The sub-boundary network formed by dense dislocation walls separates many subgrains in the original grain. With the continuation of deformation, these boundaries with low misorientation continue to absorb dislocations, and progressive lattice rotation takes place from the subgrains adjacent to the original grain boundaries. New grains are formed as the low-angle grain boundaries are transformed into high-angle grain boundaries. Eventually, the original microstructure is replaced by the new grain structure which is gradually produced from outside to inside.

Being a kinetic energy penetration weapon, the larger aspect ratio to improve the penetration performance and the geometry for more aerodynamic stability has always been the goals of the rod-shaped EFP. Due to the temporal differences in the action of the shock wave generated by the blast on the microelements of the liner, it leads to gradient strains within the liner. The microstructure, which is inhomogeneous throughout the whole volume, directly affects the power performance of Ta-2.5W EFP. Rational regulation of the strain of the liner driven by the explosive detonation enables the formation of a much more uniform microstructure of the projectile under the control of CDRX, which is very important for the engineering application of Ta-2.5W alloy.

## 5. Conclusions

The microstructure of the liner before and after deformation was compared using the EBSD technique, while the temperature of the soft recovered EFP was quantified by combining with theoretical calculations. The main findings are as follows.

The microstructure of the recovered EFP consists of fine equiaxed crystals and deformed large grains, showing obvious grain refinement. Moreover, a <011> fiber texture along the axis of the EFP is formed over the whole volume during the denotation. Due to the temporal differences in the dynamic response of the liner to the shock wave, the microstructure of different areas of the recovered projectile were inhomogeneous.

The generation of refined grains involves both thermal and non-thermal processes according to the temperature calculation. The mechanism of plastic deformation of the Ta-2.5W liner at the ultra-high strain rate is CDRX, and the grain refinement is produced by progressive lattice rotation near the grain boundaries.

In the beginning period of deformation, a large number of dislocations inside the Ta-2.5W grains are rapidly annihilated and rearranged by the enhanced DRV. The sub-boundary network formed by the dense dislocation walls divides numerous subgrains within the original grain. As deformation continues, these sub-boundaries with low misorientation sustain the absorption of dislocations, and progressive lattice rotation takes place from the subgrains adjacent to the original grain boundaries. New grains are formed as the low-angle grain boundaries are transformed into high-angle grain boundaries. Eventually, the original microstructure is replaced by the new grain structure which is gradually produced from the outside to the inside.

## Figures and Tables

**Table 1:** Parameters of Johnson–Cook for Ta-2.5W [29].

Material	A (MPa)	B (MPa)	n	C	m	$T_{m}$ (K)
Ta-2.5W	238	565	0.743	0.063	1.0	3250

**Table 2:** The calculated results of temperature of Ta-2.5W EFP.

Area	*ε*	*T* (K)	*T*/*T_m_*
1	2.26~2.59	1363~1670	0.42~0.51
2	3.00~3.36	1758~2115	0.54~0.65
3	1.34~1.73	871~1186	0.27~0.36

## Data Availability

Not applicable.
